# Oropouche Virus (OROV) and Breastfeeding Safety: Analysis of Related Orthobunyaviruses for Mother-Infant Vertical Transmission in Breast Milk

**DOI:** 10.3390/v17060738

**Published:** 2025-05-22

**Authors:** David A. Schwartz, Creuza Rachel Vicente, Mija Ververs

**Affiliations:** 1 Perinatal Pathology Consulting, Atlanta, GA 30329, USA; 2 Department of Collective Health, Universidade Federal do Espírito Santo, Vitória 29047-105, Brazil; 3 Center for Humanitarian Health, Johns Hopkins Bloomberg School of Public Health, Baltimore, MD 21205, USA

**Keywords:** Oropouche fever, Oropouche virus, breastfeeding, breast milk, vertical infection, Orthobunyavirus, milk-borne transmission, maternal–infant transmission, arbovirus, pregnancy

## Abstract

The discovery that the Oropouche virus (OROV) can be transmitted vertically from an infected pregnant mother to the fetus, resulting in fetal and placental OROV infection, miscarriage, stillbirth, and congenital malformations including microcephaly, has emphasized its public health significance. Because of the importance of breastfeeding in those areas affected by the Oropouche fever outbreak, public health agencies have continued to encourage nursing among mothers who have had OROV infection or who reside or travel in endemic regions. However, the basis for this recommendation has not been stated. At the present time, there have been no reports of the OROV being transmitted from mothers having had Oropouche fever during pregnancy to their infants through breast milk. To further evaluate the potential risk of OROV transmission through breastfeeding, we have examined the peer-reviewed literature to determine if related Orthobunyavirus species infecting humans and animals are transmissible via breast milk. Bibliographic search engines, including PubMed, Scopus, and Google Scholar, were extensively reviewed using keywords, MeSH terms, and other sources cited in the articles examined. Studies investigating Orthobunyavirus species that infect humans and animals, including reassortant strains of OROV and viruses within the Simbu serogroup, were reviewed. We found that there have been no reported events of vertical transmission of any Orthobunyavirus through breast milk. Based on these results, we believe that the advantages of breastfeeding following maternal OROV infection outweigh any negligible risk for vertical transmission.

## Communication

The Oropouche fever outbreak in Latin America and the Caribbean continues to affect pregnant women, but there has been no research investigating the transmissibility of Oropouche virus (OROV) during breastfeeding. Since 2022, there has been a rapid expansion of OROV transmission areas, with reassortant viral strains extending from the Amazon basin into different Brazilian states, Latin American countries, and Caribbean countries previously free of OROV infection cases. Additionally, OROV has spread globally through travel to Europe, the United States, and Canada [1,2]. In Brazil, during the first 3 months of 2025, there have been 7320 confirmed cases of Oropouche fever [3]. Since PAHO issued an epidemiological alert on 17 July 2024 regarding the association of OROV with vertical transmission, miscarriage, fetal deaths, and congenital anomalies [4], it has been confirmed that the virus is present in the placenta, body fluids, and tissues of some infected fetuses and newborns [5,6,7]. In a recent Brazilian study, 73 pregnant women with confirmed OROV infection were followed, with some completed pregnancies having placental infection, congenital transmission, spontaneous abortion, and central nervous system abnormalities [8]. A new syndrome of congenital OROV infection has been described [6].

Breastfeeding is a necessity in most regions where the Oropouche fever outbreak is occurring. The American College of Obstetricians and Gynecologists currently states, “It is unknown whether OROV can be transmitted through human milk. However, given the benefits of breastfeeding or chestfeeding, it is still recommended in individuals with OROV infection.” [9]. The Centers for Disease Control and Prevention similarly encourages mothers with Oropouche fever and those who travel to transmission areas to breastfeed [10]. However, neither organization has shared the scientific basis for these recommendations.

To evaluate the safety of breastfeeding with an OROV infection, we examined the peer-reviewed literature using various combinations of keywords for evidence that OROV and related arboviruses were transmissible through breastfeeding and milk in both humans and other mammals. We utilized bibliographic search engines, including PubMed, Scopus, and Google Scholar, as well as researched references within identified articles. Our search included sources from 1966 to present. Keywords included breastfeeding, breast milk, milk borne, nursing, suckling, *Orthobunyavirus*, *Bunyaviridae*, vertical transmission, mother–infant infection, lactation, neonatal viral infection, Simbu serogroup, *Bunyavirales*, and the names of individual viruses. MeSH terms included pregnancy, female, Simbu virus, Oropouche, Orthobunyavirus, abortion, milk, human, stillbirth, mothers, breastfeeding, placenta, breast, *Bunyaviridae*, *Orthobunyavirus*, *Bunyavirales* infections, fetus, fetal diseases, lactation, and communicable diseases.

OROV is a member of the genus *Orthobunyavirus*, a large group of RNA viruses with approximately 170 species exhibiting genomic rearrangement that are transmitted by hematophagous arthropods. Although some orthobunyaviruses infect humans, the vast majority have other mammalian hosts, including ruminants, marsupials, and rodents.

Orthobunyaviruses that are human pathogens include La Crosse (LACV), Keystone (KEYV), Bunyamwera (BUNV), Jamestown Canyon (JCV), Tete (TETEV), Batai (BATV), Ngari (NRIV), Maguari (MAGV), Bwamba (BWAV), California encephalitis (CEV), and group C viruses. A detailed review of the literature reveals no evidence that these viruses are transmissible by breastfeeding in human mother–infant dyads.

Reassortant viruses derived from OROV include the Iquitos virus (IQTV), the Perdões virus (PDEV), the Madre de Dios virus (MDDV), and possibly the Jatobal virus (JATV) [2]. Our analysis also found no reports of mother-to-infant transmission of OROV or these related reassortants through breast milk.

OROV is also a member of the Simbu serogroup of orthobunyaviruses, many of which cause intrauterine infections, spontaneous abortions, and congenital defects in ruminants, including CNS and musculoskeletal abnormalities [11]. A literature review revealed that none of the Simbu serogroup arboviruses, including Aino (AIN), Shamonda (SHAV), Cache Valley (CVV), Akbane (AKAV), Sango (SANV), Tinaroo (TINV), Schmallenberg (SBV), Peaton (PEA), Douglas (DOUV), and Shuni (SHUV) viruses, have been identified as being able to infect breast milk in affected animals or to be transmissible by suckling, although some reduce milk yield [12,13].

Three steps are necessary for viruses to enter breast milk following maternal infection. Spread of the virus from the primary infected organs (respiratory tract, gastrointestinal tract, etc.) via the bloodstream to the mammary glands is the initial event. Viruses must then penetrate the blood–milk barrier, replicate in mammary gland epithelium, and maintain their structural stability and infectivity within the physiological environment of breast milk. Finally, milk-borne viruses must penetrate the epithelial barriers of the infant’s gastrointestinal, tonsillar, or respiratory tracts to establish a productive infection [14]. Although it is unknown whether OROV can fulfill these requirements, current medical literature shows no evidence that it can be transmitted through breastfeeding.

Breast milk has the nutrients that neonates and infants need for their growth, development, and overall health. In addition to its nutritional content and role in maternal–infant bonding, it is rich in anti-infective antibodies and other immunological components that play a key role in protecting vulnerable children from infectious diseases outside of the womb. In particular, continued breastfeeding until 24 months of age results in a 50% decrease in mortality risk from infections, a 30% decreased risk for diarrheal disease among children less than 5 years of age, and a 32% decreased risk of hospitalizations associated with respiratory diseases among children less than 2 years of age [15]. In Brazil, where most cases of OROV infection have occurred, early initiation of breastfeeding has a prevalence of 62.5%; exclusive breastfeeding in infants less than 6 months of age has a prevalence of 45.8%; and continued breastfeeding has a prevalence of 52.1% at 12–15 months of age and 35.5% at 20–23 months of age [16]. However, breast milk may also contain cell-associated viruses, vesicle-cloaked viruses, and free virions following maternal infection that pose a risk for postnatal transmission [14,17]. Thus, it is important that breastfeeding be evaluated as a possible method for vertically transmitting an emergent viral infection from an infected mother to infant.

This communication has focused primarily on the presence or absence of OROV in breast milk as a proxy for safety. However, this is only one component of a more comprehensive risk assessment framework, as there are limitations to relying solely on virological evidence to assess the safety of breast milk from OROV-infected mothers. Incorporating principles from food safety, toxicology, nutrition, and pediatrics—especially for vulnerable populations such as infants—is essential to developing evidence-based guidance. The four key steps of a food safety risk assessment are the following [18]:

(1) Hazard Identification: Our current communication presents preliminary virological evidence regarding the presence of OROV in breast milk; however, we acknowledge that further characterization of the virus’s pathogenicity in neonates and its mode of transmission are needed to fully establish its hazard potential.
(2) Dose–Response Assessment: This step is notably absent from existing literature on OROV and breast milk. There are currently no data on the viral load threshold that could trigger infection in infants via this route, which represents a major evidence gap.
(3) Exposure Assessment: Beyond detection, it is critical to understand the frequency and amount of breast milk consumption by infants from OROV-infected mothers, potential viral persistence post-expression or while in storage, and infant immune status. These are all important factors to consider when modeling realistic exposure levels.
(4) Risk Characterization: Integrating the above elements would allow for a more nuanced understanding of the actual risk posed, which in turn would guide public health recommendations and inform risk communication strategies.

We hope that this communication provides additional scientific support for the current recommendations regarding breastfeeding following OROV infection. During an outbreak of an emerging viral disease, the paradox between the protective and infective risks of breast milk must be weighed carefully. That said, there is no evidence that OROV or other *Orthobunyavirus* genus and Simbu serogroup viruses are transmitted through breastfeeding to either humans or other mammalian hosts. Because of the overwhelming benefits that breastfeeding provides, especially in those areas where the Oropouche fever outbreak is occurring, we believe that the advantages of breastfeeding following maternal OROV infection outweigh the risks.

However, the public health community should not be complacent regarding the safety of breastfeeding following OROV infection. Understanding how viruses in general, and arboviruses in particular, disseminate to female mammary glands and are excreted in breast milk is vastly understudied [19]. The current outbreak should be considered a call to action for research into the safety of breast milk following this and other arboviral infections.

## References

[1] Schwartz D.A., Dashraath P., Baud D. (2024). Oropouche virus (OROV) in pregnancy: An emerging cause of placental and fetal infection associated with stillbirth and microcephaly following vertical transmission. Viruses.

[2] Tilston-Lunel N.L. (2024). Oropouche virus: An emerging orthobunyavirus. J. Gen. Virol..

[3] Herriman R. (2025). Brazil Oropouche cases top 7000 in 2025. Outbreak News Today.

[4] Pan American Health Organization/World Health Organization Epidemiological Alert: Oropouche in the Region of the Americas: Vertical Transmission Event Under Investigation in Brazil–17 July 2024. https://www.paho.org/en/documents/epidemiological-alert-oropouche-region-americas-vertical-transmission-event-under.

[5] Schwartz D.A. (2025). Novel reassortants of Oropouche virus (OROV) are causing maternal-fetal infection during pregnancy, stillbirth, congenital microcephaly and malformation syndromes. Genes.

[6] Ribeiro B.d.F.R., Barreto A.R.F., Pessoa A., Azevedo R.d.S.d.S., Rodrigues F.d.F., Borges B.d.C.B., Mantilla N.P.M., Muniz D.D., Chiang J.O., Fraga L.R. (2025). Congenital Oropouche in humans: Clinical characterization of a possible new teratogenic syndrome. Viruses.

[7] Schwartz D.A., Baud D., Dashraath P. (2025). A potential mechanism of transplacental transmission of Oropouche virus in pregnancy. Lancet Microbe.

[8] Cola J.P., Brioschi dos Santos A.P., Lubiana Zanotti R., Dela Costa A.E.S., Del Carro K.B., Coelho L.A.L., Miranda A.E., Vincente C.R. (2025). Maternal and fetal implications of Oropouche fever, Espírito Santo State, Brazil, 2024. Emerg. Infect. Dis..

[9] American College of Obstetricians and Gynecologists Update on Oropouche Virus and Potential Effects on Pregnancy. https://www.acog.org/clinical/clinical-guidance/practice-advisory/articles/2024/08/update-on-oropouche-virus-and-potential-effects-on-pregnancy.

[10] Centers for Disease Control and Prevention (2025). Complications of Oropouche and Pregnancy. https://www.cdc.gov/oropouche/symptoms/oropouche-and-pregnancy.html.

[11] Dashraath P., Nielsen-Saines K., Schwartz D.A., Musso D., Baud D. (2024). Vertical transmission potential of Oropouche virus infection in human pregnancies. AJOG Glob. Rep..

[12] Lechner I., Wüthrich M., Meylan M., van den Borne B.H.P., Schüpbach-Regula G. (2017). Association of clinical signs after acute Schmallenberg virus infection with milk production and fertility in Swiss dairy cows. Prev. Vet. Med..

[13] Golender N., Bumbarov V., Eldar A., Zamir L., Tov B.E., Kenigswald G., Tiomkin E. (2021). Isolation of Sango viruses from Israeli symptomatic cattle. Int. J. Vet. Sci. Res..

[14] Desgraupes S., Hubert M., Gessain A., Ceccaldi P.-E., Vidy A. (2021). Mother-to-child transmission of arboviruses during breastfeeding: From epidemiology to cellular mechanisms. Viruses.

[15] Pastro D.d.O.T., Martins F.A., Ramalho A.A., Andrade A.M.d., Opitz S.P., Koifman R.J., Silva I.F.d. (2024). Continued breastfeeding in a birth cohort in the western Amazon of Brazil: Risk of interruption and associated factors. Nutrients.

[16] Boccolini C.S., Lacerda E.M.A., Bertoni N., Oliveira N., Alves-Santos N.H., Farias D.R., Crispim S.P., Carneiro L.B.V., Schincaglia R.M., Giugliani E.R.J. (2023). Trends of breastfeeding indicators in Brazil from 1996 to 2019 and the gaps to achieve the WHO/UNICEF 2030 targets. BMJ Glob. Health.

[17] Francese R., Peila C., Donalisio M., Lamberti C., Cirrincione S., Colombi N., Tonetto P., Cavallarin L., Bertino E., Moro G.E. (2023). Viruses and human milk: Transmission or protection?. Adv. Nutr..

[18] Liu B.M. (2023). History of global food safety, foodborne illness, and risk assessment. History of Food and Nutrition Toxicology.

[19] Waitt C., Gribble K., Waitt P., Imani-Musimwa P., Liang C., Ververs M. (2025). Scarcity of research on breastfeeding and Ebola diseases is placing the lives of women and infants at risk: A call to specific action. Lancet Glob. Health.

